# Colorectal cancer care in the age of coronavirus: strategies to reduce risk and maintain benefit

**DOI:** 10.2217/crc-2020-0010

**Published:** 2020-04-16

**Authors:** John L Marshall, Ronit I Yarden, Benjamin A Weinberg

**Affiliations:** 1 Hematology & Oncology, The Ruesch Center for the Cure of GI Cancers, Lombardi Comprehensive Cancer Center, Georgetown University Medical Center, Washington DC 20057, USA; 2 Colorectal Cancer Alliance, 1025 Vermont Ave., Washington DC 20005, USA; 3 Ruesch Center for the Cure of Gastrointestinal Cancers, Lombardi Comprehensive Cancer Center, Georgetown University, Washington DC 20007, USA

**Keywords:** colorectal cancer, COVID 19, treamtment modifications

We are in unprecedented times requiring us to approach our medical obligations in unprecedented ways. Cancer care is not elective and therefore must be maintained throughout the COVID-19 pandemic [1]. However, we recognize that there is early evidence based on small studies from China [2] and Italy [3] that cancer patients are at increased risk of severe illness if they are to develop a COVID-19 infection and there is even some evidence to suggest that healthcare providers are at increased risk, possibly due to exposure to larger viral loads and repeated exposure [4,5].

Behind the scenes there are ongoing, organic collaborations among medical providers and scientists around the world working to help us endure the pandemic, but more importantly, helping us to fight back through development of treatments, prognostic and predictive markers and minimizing infections. Unpublished Data and metrics assessing the outbreak permeate across social media. New data is emerging so quickly that it is often difficult to determine our best course.

Should we continue to treat our patients using standard guidelines or should we modify our treatments to mitigate risk? In so doing, are we trading off short-term risk for ultimate worse long-term outcomes? We have already seen many instances in our own practice where we have felt forced to modify standard treatments to optimize the balance between the effective delivery of cancer care and minimize the risk to our patients and frankly to ourselves.

Many disease groups, including those representing breast cancer and melanoma, have published treatment guidelines attempting to standardize treatment modifications for the months ahead [6–8]. Individual cancer centers have developed internal guidelines for colon cancer management, but no major national group has published any recommendations to date. In partnership with the Colorectal Cancer Alliance (DC, USA) and the Otto J Ruesch Center for the cure of gastrointestinal cancers at the Georgetown University Lombardi Comprehensive Cancer Center (DC, USA), we are presenting a practical set of guidelines and recommendations for the management of colorectal cancer during the COVID-19 pandemic.

It is virtually impossible to detail every possible scenario that clinicians and patients could face over the coming months, so instead we are providing a broader outline of the basic principles we are incorporating into decision making, specific ideas of how to modify common treatment regimens and a table highlighting strategic guidance to consider when making multidisciplinary recommendations. We assume our readers will incorporate important clinical variables such as MSI-High, right-versus left-sided disease, *RAS/BRAF* and *HER 2* into individual patient decisions.

Basic principles:

-  Avoid clinic and hospital exposure
  While this may be highly variable depending on the nature of an individual healthcare practice, we know that there will continue to be an increasing number of COVID-19-positive individuals in and around hospitals. It is increasingly likely that our medical staff will become infected and, despite masks, gloves and other personal protective equipment, given the high rate of asymptomatic carriers, we want to minimize the chances of our patients becoming infected. In our way of thinking, every trip to the clinic or hospital carries some risk. We must do everything we can to reduce this controllable variable;
  We are all rapidly evolving to replace face-to-face encounters with remote, ‘telemedicine’ visits. Although embraced positively by both our staff and our patients, we recognize that we lose an important element of the human touch and confirmatory diagnostic abilities that come from a directed physical examination. But for the vast majority of our patient encounters, this technology has proven to be an effective bridge. Indeed, it is likely that these visits will permanently replace in-person visits in some circumstances, particularly long-term follow-up appointments;
  However, we have not replaced the need for in-person visits for those patients who are on intravenous therapies. Somewhat unique to colorectal cancer, we prescribe home intravenous 5-fluorouracil (5-FU) as a routine, but other agents such as oxaliplatin, irinotecan, bevacizumab, anti-EGFR agents and others, are still administered in an infusion unit by skilled nurses and supported by expert pharmacists. These agents are important to improve outcomes for our patients. Therefore, we must weigh the importance of these drugs versus the risk of infection;
-  Maintain optimal clinical outcomes, especially in the curative setting.
  Our current guidelines are based on a series of clinical trials and practice patterns, which are brought together as recipes for optimized clinical outcomes. It is important to recognize that while our current guidelines do reflect the current standards, there are significant modifications that can be made, which are unlikely to result in any major significant negative impact on an individual patient;
  However, there are key moments in the treatment of colorectal cancer where we are delivering curative therapy. Certainly, in the adjuvant setting for stage II and stage III patients, chemotherapy has a significant curative impact. We would suggest that curative therapies can still be modified without compromising long-term survival. In the metastatic setting, our treatment is primarily palliative and while we cannot simply put this treatment on hold for the next few months, we can make significant modifications that are unlikely to have a major negative impact on patient outcomes;
- Reduce myelosuppression.
  In colorectal cancer, we are increasingly recommending regimens that generate regular myelosuppression: FOLFOXIRI;
- Trifluridine-tipiracil, and other regimens are associated with high rates of grade 3–4 neutropenia and anemia [9,10]. While there is a little hard evidence that being myelosuppressed when exposed to COVID-19 will increase one’s risk of infection, we feel it is prudent to avoid low counts if at all possible. Unlike influenza, secondary bacterial pneumonias do not appear to be as commonly associated with COVID-19 but are still seen in a minority of patients [11]. Lymphopenia is frequently seen in COVID-19, but this likely reflects active viral infection, more than vulnerability to becoming infected. Nevertheless, we recommend modifying regimens to reduce myelosuppression where possible, particularly in the palliative setting;
- Avoid grade 3–4 toxicity that would require emergency room (ER) visits or hospitalizations
  Many of our treatments induce nausea, vomiting, diarrhea, mucositis and febrile neutropenia, among others, which result in emergency room evaluations and often admissions. While many of these toxicities can be predicted, most are not. Careful monitoring of kidney and liver function can help predict risk. In addition, pharmacogenetic tests of germline mutations in *DPYD*, *UGT1A1* and others, can identify rare patients at risk for significant toxicities, but these are unlikely to significantly reduce unexpected adverse events and are not recommended in standard guidelines [12];
  We do know that by lowering doses we can reduce the frequency of grade 3–4 toxicities and, fortunately in colorectal cancer, there is little evidence that dose intensity carries a survival advantage. Therefore, it would be our recommendation for those patients that are being continued on more intensive regimens that dose modifications by as much as 25% be made proactively, particularly in the first few cycles, to ensure that grade 3/4 toxicities do not emerge. Moreover, we would favor adding prophylactic growth factor support in patients with borderline neutrophil counts at baseline. We would caution using growth factor in patients with confirmed COVID-19 given the risk of capillary leak syndrome;
- Planning for 2–3 months, not a few weeks.
  While there is no specific agreement as to the timeline ahead, it is likely that the impact of the COVID-19 pandemic will be felt for the next several months. Therefore, as you are making plans with your individual patients, we recommend that you consider that the current situation will be in place for several months.

Modification ideas:

- Drop the bolus 5-FU.
  There is little consistent evidence that the bolus 5-FU portion of a FOLFOX or FOLFIRI regimen adds significant benefit. There is clear evidence that it adds toxicity in the form of myelosuppression, mucositis, and diarrhea [13]. There is controversy as to whether the leucovorin should be maintained if the bolus is dropped. To reduce the time in the infusion unit and possibly minimize toxicity further, we also recommend dropping the leucovorin;
- Change intravenous 5-FU to oral capecitabine.
  In virtually every clinical scenario, from adjuvant therapy to metastatic treatment to concurrent chemoradiation, capecitabine has proven to be equal or superior to iv. 5-FU [14–16]. To simplify dosing and schedules, we recommend continuous dosing of 1000–1500 mg by mouth twice daily, Monday through Friday. Other modifications of standard dosing include a 7-day on, 7-day off regimen. Capecitabine given at full doses, either alone or in combination with intravenous chemotherapy, is associated with more mucositis and diarrhea and should therefore, be avoided;
- Skip cycles of treatment.
  As outlined above, we are planning for several months of modified treatments. Therefore, skipping a single cycle is unlikely to have a major impact apart from the patient avoiding a trip to the hospital. Certain therapies such as bevacizumab when given as maintenance or pembrolizumab when given as chronic therapy due to their long half-lives could be skipped for a month or more. Delaying a treatment from every 2–3 weeks could have an impact over a few months and therefore should be considered when possible;
- Drop the iv. portion of a regimen and just maintain with capecitabine or other oral medications.
  We must remember that the addition of oxaliplatin to adjuvant therapy adds only a relatively small incremental improvement over 5-FU/LV or capecitabine [17]. Certainly, patients initiating adjuvant chemotherapy for high risk disease should be offered doublet chemotherapy, but one could justify starting with single agent oral therapy and adding oxaliplatin later, depending on the impact of the pandemic. While the impulse to delay adjuvant chemotherapy is enticing, data demonstrates that delaying initiation of adjuvant chemotherapy leads to inferior survival outcomes [18]. In the metastatic setting, maintenance therapy has been firmly established and can be initiated as soon as after 2–3 months of induction therapy. Standard maintenance therapy includes capecitabine with or without bevacizumab and, single-agent capecitabine should be considered. Treatment holidays or oral therapies could be used as a bridge to surgeries that are planned;
- Manage orals using telemedicine visits and outside labs.
  Supported with regular nursing education and remote visits, it is appropriate to manage patients on most oral regimens without in-person clinic visits. Most regimens do require regular laboratory testing and we would recommend referring patient’s to neighborhood providers such as LabCorp (NC, USA) and Quest (NJ, USA) for these labs instead of visits to the clinic or hospital;
- Spread out mediport flushes to 6–8 weeks.
  Many of our patients are used to the routine of having their Mediport flushed every month. If there is no other reason for the patient to come to the hospital, we recommend extending this to 6–8 weeks;
- Short-course radiation when possible.
  In the USA, the standard approach for preoperative radiation for rectal cancer is a treatment that is delivered daily over 5–6 weeks. When appropriate, we would recommend that short-course radiation be used for neoadjuvant treatment of rectal cancer [19,20]. Newer technologies including stereotactic radiosurgery have enabled much shorter treatment schedules and we would recommend using these techniques for palliative radiation where available;
- Consider ctDNA for adjuvant decision making.
  Circulating tumor DNA technologies have burst onto the colorectal scene in the last year. Primary surgery for colorectal cancer will continue over the course of the next few months and patients will be looking to us for adjuvant therapy decisions. If available, we recommend ctDNA testing to assist in adjuvant therapy decision making, by detecting minimally residual disease. Some companies providing this technology are offering in-home sample collection in support of this process. While not definitive or established, a positive tumor DNA test would compel us to initiate chemotherapy even during the pandemic. A negative test will be more difficult to apply but could justify delaying treatment or being less aggressive. Similarly, Immunoscore^®^ quantifies immune cell infiltration in resected colorectal cancer and if a patient had a high-risk stage II colorectal cancer but with high Immunoscore, adjuvant chemotherapy has a lower likelihood of benefit and should be avoided [21];
- Delay surgeries when appropriate (may be upwards of 2–3 months).
  Over the course of the next few months, many of our patients will come up on the time when their surgery was planned as part of an overall multidisciplinary strategy. This could be surgery to remove their primary tumor, a metastasectomy or the long awaited re-anastomosis. Decisions to delay surgery must be based on a multidisciplinary discussion, but could be justified based on hospital and ICU resources and patient risk. Some rectal cancer studies indicate that a watch-and-wait approach for patients with a clinical complete response to neoadjuvant chemoradiation is safe and leads to similar outcomes compared with patients who undergo surgery; however, other studies highlight an increased rate of distant metastases in those forgoing surgery [22,23]. Regardless, a watch-and-wait approach may be appropriate in order to avoid surgery and its resulting hospitalization during the pandemic. Likewise, patients scheduled for resection of metastases could be maintained on oral chemotherapy or even treatment holiday until such time that it is safe to have surgery. These delays in surgery are unlikely to have a major negative impact on a given patient apart from the anxiety of waiting, which should not be minimized. In fact, any change in therapy which is recommended solely due to COVID-19 will cause some level of anxiety among our patients. Extra time will be required to explain the rationale for the recommendation and the establishment of a revised plan that is mutually agreed upon. During this period, more will be expected of our patients and patient selection, patient resources and caregiver support are critical to optimized outcomes.

**Table 1.** Treatment modification strategies for CRC during the COVID-19 pandemic.

Treatment setting/modality	Treatment that should be commenced if possible	Treatment should not be commenced without justification	Treatment should not be stopped without justification	Treatment can potentially be stopped or delayed after careful consideration
Adjuvant	– Rectal cancer, neo-adjuvant chemo – 5FU/Oxalipatin stage III, 4–8 weeks post op	Oxaliplatin for stage II crc, cape OK	Adjuvant 5FU/cape for stage III (month 1–3)	– Oxaliplatin in adjuvant – Adjuvant after 3 months – Routine follow-up labs and scans – Routine colonoscopy
Metastatic	Front line met CRC		Induction chemo for met resection	– Maintenance therapy – Palliative chemo – Follow-up scans if stable
Surgery	– Primary resections – Obstruction, severe bleeding	Elective liver or other met resections		– Elective liver or other met resections – Rectal resections after major neo-adjuvant response
Radiation	Palliative RT	Rectal chemo/RT	Rectal chemo/RT	– Post Op RT – Palliative RT if pain controlled

CRC: Colorectal cancer; RT: Radiotherapy.

The following table was developed not as absolute guidelines but more as a framework for thinking through an individual patient’s treatment during the COVID-19 pandemic. Emphasis should continue to be placed on multidisciplinary discussions balancing the patient’s individual benefit from a given therapy against potential exposures within the hospital setting and limited resources within many hospitals dealing with a surge in COVID-19 patients.

As stated, treatment for colorectal cancer is not elective and therefore cannot simply be canceled for the next few months. We must continue to support our patients so that they receive optimized treatment for their colorectal cancer, while at the same time minimizing their individual risk of infection. We hope this review will prove useful to you as you are making individual patient decisions.

## References

[1] Zhu N, Zhang D, Wang W A novel coronavirus from patients with pneumonia in China, 2019. N. Engl. J. Med. 382(8), 727–733 (2020). 31978945 10.1056/NEJMoa2001017 PMC7092803

[2] Liang W, Guan W, Chen R Cancer patients in SARS-CoV-2 infection: a nationwide analysis in China. Lancet Oncol. 21(3), 335–337 (2020). 32066541 10.1016/S1470-2045(20)30096-6 PMC7159000

[3] Lambertini M, Toss A, Passaro A Cancer care during the spread of coronavirus disease 2019 (COVID-19) in Italy: young oncologists’perspective. ESMO Open 5, e000759 (2020). 32229501 10.1136/esmoopen-2020-000759 PMC7174009

[4] McMichael TM, Currie DW, Clark S Epidemiology of COVID-19 in a long-term care facility in King County, Washington. N. Engl. J. Med. (2020) (Epub ahead of print). 10.1056/NEJMoa2005412 PMC7121761 32220208

[5] Liu Y, Yan LM, Wan L Viral dynamics in mild and severe cases of COVID-19. Lancet Infect. Dis. (2020) (Epub ahead of print). 10.1016/S1473-3099(20)30232-2 PMC7158902 32199493

[6] Coles CE, Aristei C, Bliss J International guidelines on radiation therapy for breast cancer during the COVID-19 pandemic. Clin. Oncol. 32(5), 279–281 (2020). 10.1016/j.clon.2020.03.006 PMC7270774 32241520

[7] Consortium TC-PBC. Recommendations for prioritization, treatment, and triage of breast cancer patients during the COVID-19 pandemic: executive summary (2020). www.nccn.org/COVID-19/pdf/The_COVID-19_Pandemic_Breast_Cancer_Consortium_Recommendations_EXECUTIVE_SUMMARY.pdf 10.1007/s10549-020-05644-z PMC7181102 32333293

[8] National Comprehensive Cancer Network. Short-term recommendations for cutaneous melanoma management during COVID-19 pandemic (2020). www.nccn.org/COVID-19/pdf/Melanoma.pdf

[9] Loupakis F, Cremolini C, Masi G Initial therapy with FOLFOXIRI and bevacizumab for metastatic colorectal cancer. N. Engl. J. Med. 371(17), 1609–1618 (2014). 25337750 10.1056/NEJMoa1403108

[10] Mayer RJ, Van Cutsem E, Falcone A Randomized trial of TAS-102 for refractory metastatic colorectal cancer. N. Engl. J. Med. 372(20), 1909–1919 (2015). 25970050 10.1056/NEJMoa1414325

[11] Huang C, Wang Y, Li X Clinical features of patients infected with 2019 novel coronavirus in Wuhan, China. Lancet 395(10223), 497–506 (2020). 31986264 10.1016/S0140-6736(20)30183-5 PMC7159299

[12] National Comprehensive Cancer Network. NCCN guidelines version 2.2020 colon cancer (2020). www.nccn.org/professionals/physician_gls/pdf/colon.pdf

[13] Fuchs CS, Marshall J, Mitchell E Randomized, controlled trial of irinotecan plus infusional, bolus or oral fluoropyrimidines in first-line treatment of metastatic colorectal cancer: results from the BICC-C Study. J. Clin. Oncol. 25(30), 4779–4786 (2007). 17947725 10.1200/JCO.2007.11.3357

[14] Hofheinz RD, Wenz F, Post S Chemoradiotherapy with capecitabine versus fluorouracil for locally advanced rectal cancer: a randomised, multicentre, non-inferiority, Phase III trial. Lancet Oncol. 13(6), 579–588 (2012). 22503032 10.1016/S1470-2045(12)70116-X

[15] Van Cutsem E, Twelves C, Cassidy J Oral capecitabine compared with intravenous fluorouracil plus leucovorin in patients with metastatic colorectal cancer: results of a large Phase III study. J. Clin. Oncol. 19(21), 4097–4106 (2001). 11689577 10.1200/JCO.2001.19.21.4097

[16] Twelves C, Wong A, Nowacki MP Capecitabine as adjuvant treatment for stage III colon cancer. N. Engl. J. Med. 352(26), 2696–2704 (2005). 15987918 10.1056/NEJMoa043116

[17] Andre T, de Gramont A, Vernerey D Adjuvant fluorouracil, leucovorin, and oxaliplatin in stage II to III colon cancer: updated 10-year survival and outcomes according to BRAF mutation and mismatch repair status of the MOSAIC Study. J. Clin. Oncol. 33(35), 4176–4187 (2015). 26527776 10.1200/JCO.2015.63.4238

[18] Biagi JJ, Raphael MJ, Mackillop WJ, Kong W, King WD, Booth CM Association between time to initiation of adjuvant chemotherapy and survival in colorectal cancer: a systematic review and meta-analysis. JAMA 305(22), 2335–2342 (2011). 21642686 10.1001/jama.2011.749

[19] Swedish Rectal Cancer T, Cedermark B Improved survival with preoperative radiotherapy in resectable rectal cancer. N. Engl. J. Med. 336(14), 980–987 (1997). 9091798 10.1056/NEJM199704033361402

[20] Ngan SY, Burmeister B, Fisher RJ Randomized trial of short-course radiotherapy versus long-course chemoradiation comparing rates of local recurrence in patients with T3 rectal cancer: trans-tasman radiation oncology group trial 01.04. J. Clin. Oncol. 30(31), 3827–3833 (2012). 23008301 10.1200/JCO.2012.42.9597

[21] Galon J, Hermitte F, Mlecnik B Immunoscore clinical utility to identify good prognostic colon cancer stage II patients with high-risk clinico-pathological features for whom adjuvant treatment may be avoided. J. Clin. Oncol. 37(Suppl. 4), (2019).

[22] Maas M, Beets-Tan RG, Lambregts DM Wait-and-see policy for clinical complete responders after chemoradiation for rectal cancer. J. Clin. Oncol. 29(35), 4633–4640 (2011). 22067400 10.1200/JCO.2011.37.7176

[23] Smith JJ, Strombom P, Chow OS Assessment of a watch-and-wait strategy for rectal cancer in patients with a complete response after neoadjuvant therapy. JAMA Oncol. 5(4), e185896 (2019). 30629084 10.1001/jamaoncol.2018.5896 PMC6459120

